# Reproducibility of orthostatic blood pressure measurements in a middle-aged population-based cohort: a one-year follow-up study

**DOI:** 10.1007/s10286-026-01205-4

**Published:** 2026-05-09

**Authors:** Christian Zambach, Sofia Gerward, Steven van Zanten, Frederik J. de Lange, Artur Fedorowski, Gunnar Engström, Viktor Hamrefors

**Affiliations:** 1 Department of Clinical Sciences, Clinical Research Center 60:13, Lund University, Box 50332, 20313 Malmö, Sweden; 2 Department of Internal Medicine, Skåne University Hospital, Lund, Sweden; 3 Department of Cardiology, Reinier de Graaf Gasthuis, Delft, The Netherlands; 4 Department of Clinical and Experimental Cardiology, Heart Centre, Amsterdam Cardiovascular Sciences, Amsterdam UMC–University of Amsterdam, Amsterdam, The Netherlands; 5 Department of Cardiology, Karolinska University Hospital–Karolinska Institutet, Stockholm, Sweden; 6 Department of Medicine, Karolinska Institutet, Stockholm, Sweden; 7 Department of Cardiology, Skåne University Hospital, Malmö, Sweden

## Introduction

Orthostatic hypotension (OH) is a significant health problem in the general population, affecting approximately 30% of individuals over 70 years of age [1]. While specific reversible causes exist—such as excessive antihypertensive therapy, anemia, fatigue, or dehydration, OH often indicates cardiovascular (CV) autonomic dysfunction [2].

Data on the reproducibility of orthostatic blood pressure (BP) measurements over time remain limited. Most previous studies examined short time periods within hours or days [3, 4], and long-term test–retest correlations for orthostatic reactions in the general population are not well characterized.

We aimed to investigate the reproducibility of single hemodynamic measurements after one year in middle-aged generally healthy subjects from the general population.

## Methods

### Study population

The Swedish CardioPulmonary bioImage Study (SCAPIS) is a collaborative study between six Swedish universities and university hospitals. Between 2013 and 2018, 30,154 subjects aged 50–64 years from the general population were invited by letter to participate [5]. Orthostatic blood pressure reaction (OBPR) measurements were performed in 6000 subjects examined at the Malmö site.

For this study, 142 generally healthy participants were randomly selected for reexamination eleven to thirteen months after baseline. Assessments included anthropometrics, hemodynamic measurements (supine systolic and diastolic BP [SBP and DBP, respectively], resting heart rate (RHR), OBPRs), and updated information on antihypertensive medication. Identical protocols were applied at both examinations.

Participant consent and ethical approval were reported previously [5].

### Orthostatic blood pressure measurements

Orthostatic blood pressure reactions at baseline and reexamination were calculated by subtracting the BP after three minutes of standing up from the blood pressure measured after five minutes resting in the supine position, as follows: OBPR = supine BP_5 min_ – standing BP_3 min_ [1].

OH was defined as a decrease in SBP and/or DBP ≥ 20/10 mmHg upon standing [1].

Details on BP measurement protocols and risk factor examinations have been described in a previous publication [5].

### Statistical analysis

Orthostatic hypotension prevalence was compared at baseline and reexamination. Pearson’s correlation and two-way mixed-effect intraclass correlation coefficients (ICC) were calculated to assess agreement between baseline and reexamination values for SBP and DBP, RHR, and systolic and diastolic OBPRs (sOBPR, dOBPR, respectively). Similar analyses for the one-year measurements of C-reactive protein (CRP), creatinine, and triglycerides were performed to compare the one-year reproducibility of orthostatic BP measurements with those of other CV risk factors.

Statistical significance was set at *p* < 0.05. Analyses were performed using IBM SPSS Statistics V.28 (IBM Corp., Armonk, NY, USA).

## Results

The mean age (standard deviation) of the generally healthy study population (*n* = 142) was 57.3 (3.8) years (47.2% female). The mean body mass index (BMI) was 27.4 (3.6). Diabetes, smoking, and antihypertensive treatment prevalence were 5.6%, 14.2%, and 19.7%, respectively.

Pearson’s correlation and ICC coefficients for baseline versus one-year reexamination were SBP 0.781 and 0.781 (both *p* < 0.001); DBP 0.721 and 0.768 (both *p* < 0.001); RHR 0.736 and 0.733 (both *p* < 0.001); sOBPR 0.414 and 0.411 (both *p* < 0.001); and dOBPR 0.241 and 0.224 (both *p* = 0.04). For comparison, correlations were 0.442 for CRP, 0.858 for creatinine, and 0.704 for triglycerides.

**Fig. 1:**
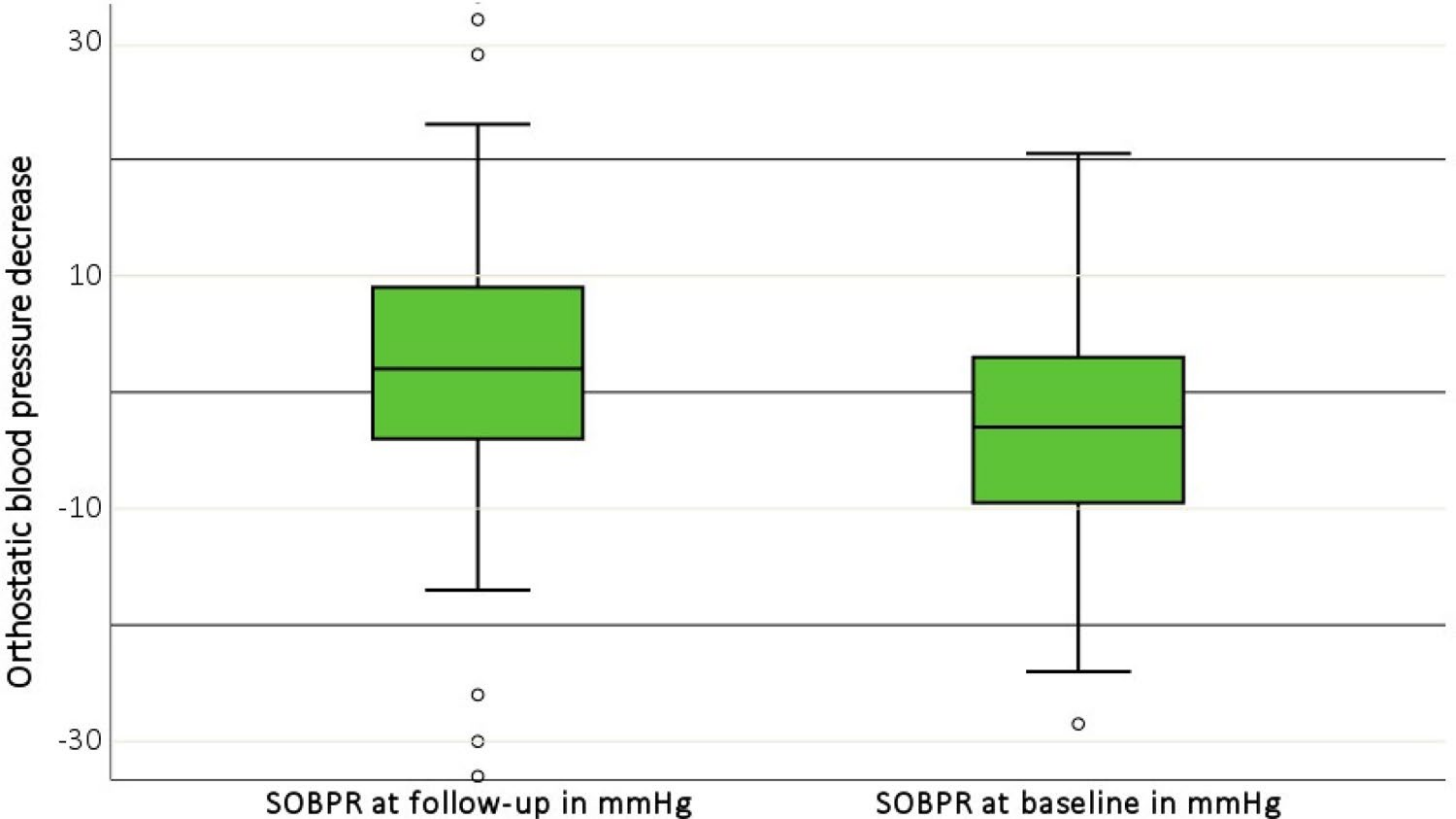
Boxplot of the distribution of systolic orthostatic blood pressure reactions (*SOBPR*) at baseline and at the 1-year reexamination

At reexamination, nine participants (6.3%) met OH criteria, none of whom had OH at baseline. Conversely, one participant had OH at baseline but not at reexamination. Figure 1 depicts sOBPR distribution at both timepoints.

## Discussion

In this generally healthy middle-aged cohort, we demonstrated good reproducibility of supine BP and heart rate measurements after one year, consistent with results from previous studies [6–8]. However, reproducibility was modest for sOBPR and poor for dOBPR, aligning with findings from earlier studies in older age-groups [2–4].

The Irish Longitudinal Study of Ageing (TILDA) provided highly relevant comparative data using continuous BP measurements in 2900 elderly participants [2]. That landmark study identified eight distinct orthostatic hemodynamic patterns, including sustained OH, transient OH, and various recovery patterns, with overall poor reproducibility at the four-year reexamination. This detailed analysis demonstrated the longitudinal dynamic nature of orthostatic BP reactions across different clinical phenotypes. Notably, certain patterns observed in particularly frail patients—presumably with more pronounced autonomic dysfunction—showed better preservation of their morphological characteristics over time [2]. Our middle-aged generally healthy population was younger and healthier than the TILDA cohort, yet we observed similarly modest reproducibility, suggesting that orthostatic variability is substantial in relatively healthy individuals.

Most previous studies employed shorter follow-up periods than our one-year interval. One previous study in twenty-eight healthy older individuals (65–75 years) found high reproducibility across multiple time points up to one year. However, that population excluded individuals with known cardiovascular disease (CVD) and/or antihypertensive treatment, and smokers, likely explaining the superior reproducibility through reduced hemodynamic variation and use of prolonged tilt-test monitoring (up to 20 minutes) [9].

### Clinical and research implications

Our findings in this generally healthy cohort have important implications for both clinical practice and research design. The reproducibility of sOBPR and dOBPR is comparable to other routinely assessed CV biomarkers, such as CRP and triglycerides, which showed similar modest reproducibility.

None of the nine subjects meeting OH criteria at reexamination had OH at baseline and only one was taking antihypertensive medication at reexamination.

Reproducibility may depend on measurement timing as OH is typically more pronounced in the morning following prolonged bed rest when cortisol levels are lower [3] and hydration status may be suboptimal. Unfortunately, we lacked data on measurement timing and prior food intake.

We recommend repeating orthostatic BP measurements to confirm OH diagnosis rather than relying on single measurements, particularly for diastolic responses. OH should be measured repeatedly with new complaints and at every outpatient visit in at-risk individuals. In patients with symptoms highly suggestive of OH, a presumptive diagnosis may be appropriate even without positive measurements [10] and verified subsequently. For research, we recommend following European Society of Cardiology (ESC) guidelines [11] and compensating for modest reproducibility by using adequately large study populations to account for both the low prevalence and measurement variability in generally healthy cohorts.

Regarding OH diagnosis, current guidelines consider a DBP decrease ≥ 10 mmHg to be significant [11]. However, one study showed that 95% of OH diagnoses relied on sOBPR alone, while isolated diastolic reactions constituted less than 6% [12]. The poor reliability of dOBPR and its lesser diagnostic role suggest that DBP may be less important for assessing OH and orthostatic intolerance.

### Limitations

Our study has several limitations. The sample size was small, though randomly selected from a larger population-based cohort. Findings from this 50- to 64-year-old cohort from the general population may not generalize to other age groups or patients with higher OH and CVD prevalence. CV risk factors accumulate throughout life, and CVD typically manifests —at least subclinically—in this age group [13]. Both OH prevalence and the proportion caused by autonomic dysfunction increase after the sixth decade, rising exponentially in later life [1]. Additionally, participants were generally healthy and ambulatory, likely to introduce selection bias toward more functional individuals, potentially underestimating the true burden of OH in this age group.

Our protocol used three minute standing measurements without beat-to-beat blood pressure monitoring, which does not capture initial OH occurring within the first minute of standing. This may have missed transient early OH patterns identified in more detailed studies such as TILDA [2].

Some authors advocate using a 30-mmHg SBP decrease threshold for OH diagnosis when resting SBP ≥ 160 mmHg [14]. One subject with OH at reexamination would not meet this criterion.

Finally, we lack data on symptoms during orthostatic provocation, precluding assessment of whether the nine subjects with OH had symptomatic OH.

## Conclusions

Resting hemodynamic parameters, including supine BP and heart rate, are reliably reproducible after one year in the general population. In contrast, the reproducibility of single orthostatic BP measurements in generally healthy middle-aged individuals is modest for systolic responses and poor for diastolic responses, comparable to traditional biomarkers such as CRP. Single measurements should be interpreted cautiously, and repeated measurements should be considered for diagnosing OH. For studies involving orthostatic BP measurements in generally heathy populations, we recommend compensating for low prevalence and modest reproducibility by using adequately large study populations.

## Data Availability

The datasets generated during and/or analyzed during the current study are not publicly available due to regulations/IRB but are available from the corresponding author on reasonable request.

## Abbreviations

BP: Blood pressure
CI: Confidence interval
CRP: C-reactive protein
CV: Cardiovascular
CVD: Cardiovascular disease
DBP: Diastolic blood pressure
dOBPR: Diastolic orthostatic blood pressure reactions
ESC: European Society of Cardiology
ICC: Intraclass correlation coefficient
OH: Orthostatic hypotension
OBPR: Orthostatic blood pressure reactions
RHR: Resting heart rate
SBP: Systolic blood pressure
SCAPIS: The Swedish CardioPulmonary bioImage Study
SD: Standard deviation
sOBPR: Systolic orthostatic blood pressure reactions
TILDA: The Irish Longitudinal Study on Ageing
